# Cytomegalovirus: A Troll in the ICU? Overview of the Literature and Perspectives for the Future

**DOI:** 10.3389/fmed.2020.00188

**Published:** 2020-05-15

**Authors:** Jolien Schildermans, Greet De Vlieger

**Affiliations:** ^1^Clinical Division of Intensive Care Medicine, University Hospitals Leuven, Leuven, Belgium; ^2^Clinical Division and Laboratory of Intensive Care Medicine, Department of Cellular and Molecular Medicine, KU Leuven, Leuven, Belgium

**Keywords:** herpes virus, cytomegalovirus, reactivation, critical illness, immunoparalysis, sepsis

## Abstract

Cytomegalovirus (CMV) is one of the most pathogenic viruses in human. After a primary infection, CMV resides in the host for life as a latent infection. When immunity is reduced, CMV can escape the suppressive effects of the immune system and lead to viremia and antigenemia. This reactivation, first seen in transplant patients, has also been documented in non-immunocompromised CMV-seropositive critically ill patients and is associated with higher morbidity and mortality. In the latter, it is not clear whether CMV reactivation is an innocent bystander or the cause of this observed worse outcome. Two studies showed no difference in the outcome of CMV-seropositive and seronegative patients. In addition, proof-of-concept studies investigating prophylactic antiviral treatment to prevent CMV reactivation during critical illness, failed to show a beneficial effect on interleukin levels or clinical outcome. Further research is necessary to resolve the question whether CMV replication impairs the prognosis in non-immunocompromised critically ill patients. We here give a concise overview on the available data and propose strategies to further unravel this question. First, post-mortem investigation may be useful to evaluate the effect of viral replication on organ inflammation and function. Second, further research should focus on the question whether the level of viremia needs to exceed a threshold to be associated with worse outcome. Third, clinical and biochemical assessments may help to identify patients at high risk for reactivation. Fourth, preemptive treatment based upon early detection of the virus is currently under investigation. Finally, immune-stimulating biologicals may be beneficial in high-risk groups.

## Introduction

The human cytomegalovirus (CMV) is a beta herpes virus that only infects human after transmission by body fluids such as saliva, blood and urine (1). In immunocompetent hosts, a primary infection frequently passes asymptomatically, although a mononucleosis-like syndrome can develop with fever, myalgia and adenopathy (1). It can be complicated by severe organ disease as colitis, pneumonitis, hepatitis, meningitis and myocarditis and it can trigger a Guillain-Barré syndrome (1). Pathologic examination of infected tissue typically shows “owl's eye inclusions”, a pathognomonic sign of CMV infection (2).

After a primary infection, CMV establishes latency in the body, with the myeloid lineage as main reservoir (1). In developed countries, ~60–80% of the adults have been infected with CMV, and in developing countries incidences rise even to 100%. Risk factors for a latent CMV infection include age, poor socioeconomic status and female gender (3). This latent viral infection is hallmarked by presence of CMV in white blood cells, but with undetectable viral loads because the host defense system suppresses viral replication. Approximately 10% of all circulating CD4+ and CD8+ T cells are involved in this process (4). When immunity is weakened, reactivation can occur and viral antigens spread into the blood (1, 2). This reactivation has first been identified as an important cause of morbidity and mortality in transplant recipients (5) and HIV-infected patients (6); and later it has been increasingly encountered in non-immunocompromised critically ill patients. Here, we will briefly overview the current guidelines to prevent CMV reactivation in transplant recipients, and we will focus in-depth on the available data in critically ill patients.

## CMV Reactivation in Transplant Recipients

CMV is the leading viral opportunistic pathogen in immunocompromised patients. In these patients, reactivation can be asymptomatic, when viral replication is detectable without associated symptoms, and is named “CMV infection” (7). When viral replication causes symptoms, it is called “CMV disease.” This disease is subdivided in a “CMV syndrome” when the patient has fever, malaise, leukopenia and/or thrombopenia without organ disease; or in “tissue invasive disease” when organs are involved (7). CMV infection is associated with increased risk of graft failure, high morbidity and mortality, and has been named the “troll” of transplantation as “the virus lurks quietly in the shadows, posed to emerge at any moment and complicate recovery” (8). International guidelines advise to administer antiviral therapy to prevent this reactivation in solid organ and hematopoietic cell transplanted patients (7, 9). Currently, there are two preventive strategies: 1. “prophylaxis” with administration of antiviral medication to patients “at risk”; and 2. “preemptive therapy” with regular monitoring for plasma CMV viral load and administration of antiviral treatment only when a threshold is exceeded (7). This strategy offers the benefit that not all patients are exposed to toxic antiviral treatment. It is recommended to measure the viral load with polymerase chain reaction (PCR) (7). Unfortunately, a lack in standardization of various assays makes it difficult to compare results from different institutes. As from 2010, the World Health Organization published a standard for nucleic acid amplification techniques with the aim to uniform the results and treatment among different care centers (10). However, the threshold above which viral replication should be treated preemptively is still unknown and remains a matter of debate (7).

Assessing the risk for reactivation is necessary to decide which preventive strategy should be applied. This assessment includes the serostatus of donor and receptor, the transplanted organ and the immunosuppressive strategy. Lung transplant recipients and CMV-naive patients who received a CMV infected organ are at highest risk of reactivation and in those, guidelines advise to use the prophylactic strategy. Consequently, no recent data are available on the reactivation rate when no prophylaxis is administered in high-risk patients. In a mixed group of kidney, lung, liver and heart transplant recipients who received preemptive treatment, the incidence of CMV viremia and CMV disease was 48.9 and 6.9%, respectively (11).

## Critical Illness Induced Immunosuppression

For many years, sepsis-induced hyperinflammation has been blamed for the high early mortality in septic patients. Nevertheless, strategies that block this response have been unsuccessful and have even caused additional harm (12). Consequently, these treatments have been largely abandoned. Meanwhile, new insights uncovered that also anti-inflammatory adaptations are engendered and this even at the very early start of critical illness. Indeed, there is also an increase in circulating anti-inflammatory cytokines and the adaptive immune system is affected, as reflected by a reduced human leucocyte antigen DR expression, increased lymphocyte apoptosis and a reduced capacity of lymphocytes to produce cytokines (13). These alterations persist after the acute phase, and are more apparent in this prolonged phase of critical illness when this “immunoparalysis” translates into an increased susceptibility to opportunistic infections such as opportunistic bacterial infections, invasive fungal infections and reactivation of viruses. Many herpes viruses reactivate during critical illness, but CMV is associated with the worst outcome (14). Consequently, CMV reactivation during critical illness has been increasingly studied during last decades.

## CMV Reactivation in Critically ill Patients

### Incidence, Risk Factors and Outcome

More than two decades ago, Papazian reported biopsy-proven CMV pneumonitis in 29% to 50% of the intensive care unit (ICU) patients with acute respiratory failure (15–17). Since then, many observational studies have documented CMV reactivation (14, 18–42) (Table 1).

**Table 1:** Overview of the rate and timing of CMV reactivation in observational studies in critically ill patients.

	**Study design**	**Study period**	**Study population**	**Severity scores**	***n***	**% CMV IgG**	**Samples**	**Detection method**	**Reactivaton rate**	**Time to reactivation (days)**
Domart et al. (18)	Prospective observational	1981–1986	Mediastinitis after cardiac surgery	APACHE II 14.6 ± 7.3	115	–	Urine and blood	Culture	25.2%	37 ± 22
Cook et al. (19)	Retrospective case control	1989–1994	Persistent sepsis SICU	APACHE II 13.0 ± 1.3 when reactivation, 14.2 ± 0.8 when no reactivation	142	–	Blood, BAL, sputum, skin	Culture	14% (CMV and HSV)	–
Kutza et al. (20)	Prospective observational	–^a^	Sepsis		34	93.90%	Blood	pp65 and PCR	32.4%	PCR: 4 pp65: 11
Heininger et al. (21)	Prospective observational	1998–1999	SAPS II > 41 in SICU	SAPS II 42.2 ± 13.5	56	100%	Plasma, leukocytes, LRT	Culture and PCR	35.60%	10.8
Cook et al. (22)	Prospective observational	15 months^a^	SICU LOS > 5 days	APACHE II 13.1 ± 0.5	104	73.10%	Blood and LRT	Culture	15% in respiratory tract, 5.8% in blood	28 ± 4
Jaber et al. (23)	Retrospective case control	1995–2001	Fever > 72 hours	SAPS II 50 ± 16	40 and 40 controls	–	Blood	pp65	17%	20 ± 12
Von müller et al. (24)	Prospective observational	9 months^a^	Septic shock and ICU LOS ≥ 7 days	SOFA 10	25	100%	Blood	pp65	32%	–
Limaye et al. (25)	Prospective observational	2004–2006	Mixed^b^	APACHE II 21 (range 7–36)	120	100%	Plasma	PCR	33%; >1000 copies in 20%	12 (range 3–57)
Ziemann et al. (26)	Retrospective observational	2001 and 2003–2004	SICU with LOS > 14 days	–	99	73%	Plasma	PCR	35%	17.0 ± 15.3
Chiche et al. (27)	Prospective observational	2 years^a^	MICU and MV ≥2 days	SAPS II 48 ± 17 SOFA 9 (IQR 6–11)	242	80%	Blood and BAL	pp65 on blood, culture on BAL	16.10%	16 (6–25)
Chilet et al. (28)	Prospective observational	2008–2009	Surgical and trauma ICU and ICU LOS > 5 days	–	53	100%	Plasma and tracheal aspirate	PCR	39.7% (in blood 30.2%)	16.5 (0–28) in plasma
Bordes et al. (29)	Prospective observational	2008–2010	Burns, TBSA > 15%	–	29	72.40%	Blood	PCR	51.70%	13 ± 9
Heininger et al. (30)	Prospective observational	2004–2006	Severe sepsis	SAPS II 43.0 (IQR 36–51) SOFA 8.0 (IQR 7–11)	97 (86 analyzed)	100%	Plasma, leukocytes and LRT	PCR	40.7% (in blood 11.6%)	24.5 (range 0–49)
Chiche et al. (31)	Prospective case control	2008–2011	MICU and MV > 2 days	SAPS II 48 SOFA 9	15, 15 controls	100%	Blood	pp65	27%	5 (3–19)
Coisel et al. (32)	Prospective observational	1 year^a^	MICU, MV, and suspected pneumonia	SAPS II 45 (IQR 31–55)	93	77%	Blood and BAL	pp65 on blood, PCR on BAL	23.7%	–
Bravo et al. (33)	Prospective observational	2008–2009 and 2011–2012	SICU	APACHE II 21 (range 10–39) SAPS II 48 (range 23–82)	78	100%	Plasma, LRT and saliva	PCR	46%^c^	10 (range 0–34)
Osman et al. (34)	Prospective observational	3 months^a^	MV	–	51	–	Serum	PCR	68.6%	–
Walton et al. (14)	Prospective observational	2009–2013	Mixed ICU	APACHE II18 in septic and 5 in nonseptic SOFA 7 in septic, 2 in nonseptic	720	70.2%	Whole blood and plasma	PCR	24.2%	–
Al-Musawi et al. (35)	Retrospective case control	2010–2013	Mixed ICU, thrombopenia	APACHE II 21 when no reactivation 27 when reactivation	52, 47 controls	83.8%	Plasma	PCR	–	–
Frantzeskaki et al. (36)	Prospective observational	2010–2012	MV in mixed ICU	APACHE II 20 range 4–43	80	100%	Plasma	PCR	13.75%	7
Lopez Roa et al. (37)	Prospective observational	2004–2006	Mixed ICU	APACHE II median 21 (range 7–36)	115	100%	Plasma	PCR	34.0%	12 (range 3–57)
Ong et al. (38)	Prospective observational	2011–2013	ARDS and MV for at least 4 days	APACHE III 79–81	306	100%	Plasma	PCR	26.0%	–
Osawa et al. (39)	Prospective observational		BSI	APACHE II 28 when reactivation 24 when no reactivation	100	100%	Plasma	PCR	20.0%	–
Ong et al. (40)	Prospective observational	2011–2013	ARDS and MV for at least 4 days	APACHE IV 91 when reactivation 76 when no reactivation	271	100%	Plasma	PCR	27.0%	8.5
Ong et al. (41)	Prospective observational	2011–2014	Septic shock and ICU LOS > 4 days	APACHE IV 85 when reactivation 82 when no viral reactivation^d^	399	65%	Plasma	PCR	27.0%	–
Hraiech et al. (42)	Retrospective obervational	2011–2017	Severe ARDS with vvECMO ≥2 days	SAPS II 51	123	–^e^	Blood and BAL	PCR	17.9% in blood 22.0% in blood and BAL	–

a *study period not mentioned in the original manuscript*.

b *burns TBSA at least 40 or 20% and inhalation injury, TICU with ISS >15 and TF of more than 4U PC, MICU with sepsis, CICU with acute myocardial infarction*.

c *CMV reactivation includes BAL positivity without viraemia*.

d *viral reactivation includes also other herpes viridae (CMV, Epstein-Barr virus, Human herpesvirus 6, herpes simplex virus (HSV) type 1, HSV type 2, and varicella zoster virus)*.

e *estimated high by authors based upon epidemiology*.

*N, number; CMV, cytomegalovirus; IgG, antibodies; APACHE, acute physiology and chronic health evaluation II; SICU, surgical intensive care unit; BAL, broncho-alveolar lavage; HSV, herpes simplex virus; PCR, polymerase chain reaction; pp65, CMV antigen; SAPS II, simplified acute physiology score II; LRT, lower respiratory tract; LOS, length of stay; SOFA, sequential organ failure assessment; MICU, medical intensive care unit; MV, mechanical ventilation; IQR, interquartile range; TBSA, total body surface area; ARDS, acute respiratory distress syndrome; vvECMO, veno-venous extracorporeal membrane oxygenation*.

The reactivation rate depends on the studied population and on the detection method. Early reports used viral cultures, but these are less sensitive and slower than antigen and PCR testing. Antigen detection has a good sensitivity in non-leukopenic patients, but as the test is performed on white blood cells, it is not accurate when patients are leukopenic (7). Nowadays, PCR is the preferred detection method. The highest reactivation rates have been observed in patients with sepsis (21, 43), and there are several biological explanations: CMV reactivation is triggered by cytokines as tumor necrosis factor α and interleukin-1β (44), by endotoxins (45); and by endogenous and exogenous catecholamines (46). Administration of steroids is also associated with higher reactivation rates (22, 23, 40). Finally, the risk increases with higher severity of illness (40, 43).

In critically ill patients, CMV reactivation is associated with prolonged stay in the ICU and in hospital (18, 21–28, 30–33, 47), increased risk for infections (14, 22, 23, 27, 32), prolonged need for mechanical ventilation (MV) (22–24, 26, 27, 30–33), and doubled mortality (19, 22, 23, 26, 32, 34, 35, 40, 41, 43, 47) (Table 2).

**Table 2:** Overview of the outcome in CMV reactivating and non-reactivating critically ill patients and the rate of antiviral treatment in observational studies.

	**Mortality**	**ICU LOS (days)**	**Duration of MV (days)**	**Infections or sepsis**	**Antiviral treatment**
Domart et al. (18)	Higher when reactivation	69 vs. 48 *P* < 0.05	–	–	–
Cook et al. (19)	65 vs. 35% *p* < 0.01	No difference	–	–	75%
Kutza et al. (20)	No difference	–	–	–	–
Heininger et al. (21)	55 vs. 36% *p* = 0.17	30 vs. 23 *p* = 0.04	–	–	2 patients, both died
Cook et al. (22)	50 vs. 27% *p* = 0.15	40.5 vs. 18.9 *p* = 0.001	32.8 vs. 12.7 *p* < 0.001	7.9 vs. 3.5 episodes *p* = 0.0001	–
Jaber et al. (23)	50 vs. 28% *p* = 0.02	41 vs. 31 *p* = 0.04	35 vs. 24 *P* = 0.03	75 vs. 50% *p* < 0.05	–
Von müller et al. (24)	63 vs. 35% non-significant	42 vs. 18 *p* < 0.01	39 vs. 16 *p* < 0.01	50 vs. 59% not significant	No patients treated
Limaye et al. (25)	–^a^	–^a^	–	–	–
Ziemann et al. (26)	28.6 vs. 10.9% *p* = 0.048	32.6 vs. 22.1 *p* < 0.001	21.1 vs. 16.2 *P* = 0.02	–	1 patient, survived
Chiche et al. (27)	54 vs. 37% *p* = 0.082	32 vs. 12 *p* < 0.001	In survivors: 27 vs. 10 *p* < 0.001	69 vs. 33% *p* < 0.001	54%
Chilet et al. (28)	61 vs. 46% *p* = 0.40	37 vs. 11 *P* = 0.01	–	–	No patients treated
Bordes et al. (29)	20 vs. 33% *p* = 0.59	57.7 vs. 24.0 *p* = 0.06	39 vs. 10 *p* = 0.37	3.1 vs. 1.2 episodes *p* = 0.06	–
Heininger et al. (30)	37.1 vs. 35.3% *p* = 0.86	30.0 vs. 12.0 *p* = 0.02	22.0 vs. 7.5 *p* < 0.001	–	No patients treated
Chiche et al. (31)	40 vs. 13.3% *p* = 0.21	28 vs. 14 *p* = 0.01	24 vs. 8 *P* < 0.02	–	–
Coisel et al. (32)	55 vs. 20% *p* < 0.01	25.5 vs. 13.0 *p* = 0.04	19.5 vs. 10.0 *p* < 0.01	46 vs. 13% *p* < 0.01	All reactivations treated
Bravo et al. (33)	55.6 vs. 35.7% *p* = 0.11	27 vs. 10 *p* < 0.001	24 vs. 7 *p* < 0.001	–	No patients treated
Osman et al. (34)	74.3 vs. 31.1% *p* = 0.003	8.14 vs. 4.31 *p* = 0.08	–	82.9 vs. 100% *p* = 0.16	–
Walton et al. (14)	Higher 90d mortality	Almost doubled	–	Significant more fungal and bacterial infections	–
Al-Musawi et al. (35)	80.8 vs. 51.1% *p* = 0.003	103 vs. 60 *p* = 0.22	–	–	–
Frantzeskaki et al. (36)	45 vs. 27% non-significant	32 vs. 21 non-significant	27.5 vs. 18 *p* < 0.001	–	–
Lopez Roa et al. (37)	–^b^	–	–	–	–
Ong et al. (38)	–	–	–	–	–
Osawa et al. (39)	–	–	–	–	–
Ong et al. (40)	31 vs. 15% *p* < 0.01^c^	–	15 vs. 8 (*p* < 0.01) No difference when multivariable correction	–	–
Ong et al. (41)	33 vs. 23% *P* < 0.01	–	–	–	–
Hraiech et al. (42)	71 vs. 59% non-signficant	29 vs. 16 non-significant	–	–	51% patients treated

a *higher risk for continued hospitalisation or death at day 30 when CMV reactivation*.

b *death or continued hospitalisation at day 30: 45 vs. 41%, significant after multivariable analysis*.

c *difference not significant after multivariable correction*.

*ICU, intensive care unit; LOS, length of stay; MV, mechanical ventilation*.

Three observational studies reported antiviral treatment in patients with CMV reactivation (19, 27, 32), and in two of them mortality was higher in CMV reactivating than in non-reactivating patients (19, 32). In the study that treated 54% of the patients, treated patients had a non-significant higher mortality than non-treated patients (62 vs. 44%, respectively) (27). As the decision to start antiviral treatment was left to the physician's decision, it is possible that the sickest patients received antivirals more often, which may explain the higher mortality. Indeed, data from observational trials cannot resolve the question whether CMV is a pathogen or bystander of the observed worse outcome. Some experts suggest interventional trials with antiviral treatment to address this question (48–50). It has been shown that CMV replication occurs only in CMV seropositive patients (43), and as such, the presence of CMV antibodies represents a good entry criterion for interventional studies. This theory has been challenged by De Vlieger et al. (51) who investigated the association between CMV seropositivity at ICU admission and outcome. In this study, involving over 1,500 patients with an ICU stay of 3 days or more, no association was found between CMV serostatus and ICU outcome. In addition, there was no difference in ICU or hospital mortality in subgroups with prolonged ICU stay, sepsis, or higher disease severity (51). Ong et al. (38) extended these results in over 300 mechanically ventilated patients with acute respiratory distress syndrome (ARDS), as they found no association between CMV seropositivity and the number of days alive and free of mechanical ventilation (MV) on day 28. However, in a *post-hoc* defined subgroup of ARDS induced by septic shock, they found an improved outcome in CMV-seronegative as compared to seropositive patients (38). One possible explanation is a higher reactivation rate in patients with sepsis-induced ARDS as compared to the overall ARDS patients (34 vs. 27%, respectively) (40).

### Interventional Studies in Mice

In a model with cecal ligation and puncture (CLP), all CMV seropositive mice showed viral reactivation in the lungs, liver, spleen and salivary glands (52). Administration of ganciclovir to prevent this reactivation did not reduce mortality, but shortened the CLP-induced inflammatory response to a duration seen in CMV seronegative mice (53). In addition, lung biopsies showed that blocking the viral replication with antivirals resulted in less fibrosis as compared to untreated mice (53). The results of these studies gave the starting signal for interventional trials in critically ill human.

### Interventional Studies in Human

Recently, two proof-of-concept studies in ICU patients have been performed (54, 55). The Cytomegalovirus Control in Critical Care (CCCC-trial) was an interventional trial in CMV seropositive patients who were mechanically ventilated for at least 24 h. The patients received valaciclovir, valganciclovir, or no treatment. The study was stopped early because a higher mortality was observed in the intervention arms (54).

A second randomized controlled trial compared the effect of ganciclovir to placebo on the evolution of interleukin-6 levels in CMV seropositive patients with sepsis or trauma and respiratory failure (GRAIL-trial) (55). Reactivation occurred more frequently in the ganciclovir group (11.9%) than in the valganciclovir group of the CCCC trial (2.2%). This may be explained by the higher risk of the studied population, as 88% had sepsis upon admission and reactivation in blood occurred in 38.9% of the placebo-treated patients as compared to 27.3% of the non-treated patients in the CCCC trial. In the GRAIL trial, the rate of CMV replication was significantly reduced in the ganciclovir arm, but the evolution of IL-6 did not differ in the two groups (55). This primary outcome was chosen because IL-6 correlated with outcome in a *post-hoc* analysis of the ARDS network trial (56). Later, it has been shown that the evolution of IL-6 and other cytokines was similar in CMV reactivating patients and a matched cohort of non-reactivating patients, and thus may be not a good surrogate endpoint (57). Interestingly, the GRAIL trail showed that ganciclovir-treated patients had significantly more ventilator free days at 28 days than placebo-treated patients in the subgroup of sepsis-induced respiratory failure (55).

A third randomized controlled trial is investigating preemptive treatment for CMV reactivation in critically ill patients. This study has also evaluated the effect of treating herpes simplex virus oropharyngeal reactivation in mechanically ventilated patients, and found no increase in the ventilator-free days at day 60 as compared to placebo. However, there was a trend to a reduced mortality and a significant increase in the time-to-event analysis for mortality in the intervention arm (58). The results whether preemptive treatment of CMV reactivation is able to improve outcome are expected soon (NCT02152358).

## Discussion

CMV reactivation was first reported in transplant recipients, and it has been increasingly documented in critically ill patients in whom it is associated with high morbidity and mortality. Several years have passed and experts are still debating whether CMV is the culprit or a simple bystander (50, 59). CMV serology was not associated with worse outcome in a large group of patients without additional risk factors, nor in patients with sepsis, prolonged ICU stay or higher severity scores (51). Moreover, two interventional studies have shown negative results and one was even stopped early because of higher mortality in patients who received antiviral treatment (54, 55). The second study showed an increase in ventilator-free days at day 28 in patients with sepsis (55). Although this was a secondary endpoint in a subgroup, it warrants further research. Now is the time to plan an optimal strategy, as new interventional trials in the overall CMV seropositive patients will probably not solve the question and may even induce unnecessary harm. There are several considerations that we must take into account.

First, identification of subgroups at high risk for reactivation is a crucial step to select a target population for future interventional trials, as unnecessary treatment in the patients in whom reactivation does not occur is likely to cause harm without adding benefit. A metaanalysis published in 2009 showed that the rate of reactivation in CMV seropositive patients was 36% with PCR or antigen detection (43). When studies until 2016 were evaluated, reactivation occurred in 31% of the CMV-seropositive patients when PCR was used (60). Thus, CMV reactivation seems to occur less frequently in the studies that were published after 2009 even though the studied patients had additional risk factors such as sepsis or prolonged mechanical ventilation (36, 38, 40, 41). Steroids are frequently administered in septic shock and ARDS and have been identified as risk factor for reactivation (22, 23, 40). Since 2009, the use of steroids has reduced over the time, as the CORTICUS trial showed no beneficial role of steroids in septic patients (61). In addition, other strategies to reduce the hyperinflammatory response have also been abandoned (12). Consequently, it is very likely that the rate of CMV reactivation has changed over time. New, multicenter, observational trials are needed to reevaluate the current incidence of reactivation. It is unlikely that reactivation rates will reach the 49% that has been seen in preemptively treated solid organ transplant recipients (11). As such, critically ill patients have a lower risk of reactivation and will benefit less from prophylactic antiviral treatment than transplanted patients.

Second, there is no consensus definition of reactivation and while many authors report data on viremia, others include viral detection in the respiratory tract (27, 28, 30, 32, 33, 42). Further evaluation of the impact of CMV detection in respiratory samples after transplantation has also been put on the agenda as an urgent Research Topic (7).

Third, it is unknown whether the level of viremia needs to exceed a threshold to be harmful. This has also been overthought in transplant recipients, but data on the untreated history and outcome of viral replication after transplantation are lacking as prevention is generally accepted in these patients (7). In critically ill patients, Limaye et al. (25) showed that the risk for prolonged hospitalization increased when the viral load increases and Bordes et al. (29) found worse outcome in burn patients when viral load exceeded 1,000 copies per milliliter as compared to those with low viral load reactivation. Post-mortem examination may be especially helpful to evaluate whether organ damage occurs above a threshold. More than two decades ago, Papazian found CMV inclusions, a pathognomonic sign of CMV disease, in patients with acute respiratory failure (16). At that time, packed cells were not leukoreduced (62) and it is possible that those patients had a transfusion-related primary infection rather than reactivation. Indeed, recent autopsy studies in critically ill patients have not reported CMV disease in non-immunocompromised patients (63–65). While this may indicate that CMV does not invade organs, another explanation may be the short length of stay (median ICU day 2 or 3) in these autopsy studies while CMV reactivation generally only occurs later during critical illness. It would be interesting to evaluate whether CMV reactivation leads to organ disease and, if so, whether this is related to the viral load.

Fourth, as CMV reactivation is likely caused by a reduced immune response, a strategy that assesses the immunologic response may be helpful to predict reactivation. One possible marker of upcoming reactivation is the interferon-γ (IFN-γ) production by CMV-specific T lymphocytes upon exposure to CMV-antigens. Higher CMV reactivation rates have been documented in critically ill patients in whom this response was lacking (31, 66). Recently, a commercial test to measure this response has become available (CMV-QuantiFERON, Qiagen). In solid organ transplant patients, a low CMV-QuantiFERON-response was associated with a higher risk to develop CMV infection (67, 68). Few studies have evaluated the test in critically ill patients (69). Nowadays, the test is not used in clinical setting, but randomized controlled trials in solid organ transplant recipients are investigating whether this test is useful to individualize the duration of antiviral treatment.

Last, immune stimulating biologicals have been suggested to treat infectious complications in ICU patients. This strategy may be able to target all opportunistic infections that have been typically observed in these patients. In a normally functioning immune system, endogenous immune components act as control mechanisms and evaluate the immune system at several checkpoints (70). These pathways are altered in cancer and sepsis, and these alterations are associated with a worse prognosis (71). In cancer patients, interventions aiming to restore this response have shown to reduce the tumor load and are licensed for therapeutic use (70). The immunological adaptations that occur in critically ill patients have many similarities with the alterations seen in cancer patients (72). Based upon these findings, successful treatments in oncological patients may also improve outcome in critically ill patients. Products that are under investigation in sepsis and/or ARDS are granulocyte-macrophage colony-stimulating factor (73–75), anti-programmed cell death 1 antibodies, anti-programmed cell death ligand 1 antibodies (anti-PD-L1) (76), recombinant INFγ (77), and recombinant human interleukin-7 (78). Patients with CMV reactivation have been suggested good candidates for immune-enhancing therapy (12), but to the best of our knowledge there are currently no trials focusing on these patients.

In conclusion, CMV is one of the most pathogenic viruses in human, and observational reports have described the rate, risk factors and associated outcome of CMV reactivation in critically ill patients. Despite repetitively documented higher morbidity and mortality associated with viral reactivation, it remains to be elucidated whether this is association is causal. Two recent randomized controlled prophylactic trials with a proof-of-concept design were not conclusive. To our opinion, further research should first focus on identifying patients at high risk for reactivation and on the level or viremia, which may indicate patient in whom antiviral prophylaxis may affect outcome. Until then, prophylactic trials are likely to be inconclusive and may induce unnecessary harm.

## Author Contributions

JS drafted the manuscript. GD critically revised the manuscript and added important research perspectives.

## Conflict of Interest

The authors declare that the research was conducted in the absence of any commercial or financial relationships that could be construed as a potential conflict of interest.
